# Better Management of Alcohol Liver Disease Using a ‘Microstructured Synbox’ System Comprising *L*. *plantarum* and EGCG

**DOI:** 10.1371/journal.pone.0168459

**Published:** 2017-01-06

**Authors:** Praveen Rishi, Sumeha Arora, Ujjwal Jit Kaur, Kanwaljit Chopra, Indu Pal Kaur

**Affiliations:** 1 Department of Microbiology, Basic Medical Sciences Block, South Campus, Panjab University, Chandigarh, India; 2 University Institute of Pharmaceutical Sciences, Panjab University, Chandigarh, India; University of Catania, ITALY

## Abstract

Synergistic combination of probiotics with carbohydrate based prebiotics is widely employed for the treatment of various gut related disorders. However, such carbohydrate based prebiotics encourage the growth of pathogens and probiotics, equally. Aim of the study was (i) to explore the possibility of using epigallocatechin gallate (EGCG) a phenolic compound, as a prebiotic for *L*.*plantarum*; (ii) to develop and evaluate a microstructured synbox (microencapsulating both probiotic and EGCG together) in rat model of alcohol liver disease (ALD); and, (iii) to confirm whether the combination can address issues of EGCG bioavailability and probiotic survivability in adverse gut conditions. Growth enhancing effect of EGCG on *L*. *plantarum* (12.8±0.5 log _10_ units) was significantly (p≤0.05) better than inulin (11.4±0.38 log _10_ units), a natural storage carbohydrate. The formulated synbox significantly modulated the levels of alcohol, endotoxin, hepatic enzymes and restored the hepatoarchitecture in comparison to simultaneous administration of free agents. Additionally, using a battery of techniques, levels of various cellular and molecular markers viz. NF-kB/p50, TNF-α, IL12/p40, and signalling molecules TLR4, CD14, MD2, MyD88 and COX-2 were observed to be suppressed. Developed microbead synbox, as a single delivery system for both the agents showed synergism and hence, holds promise as a therapeutic option for ALD management.

## Introduction

In view of increasing demand for bioecological and nutritional control of diseases, probiotics alone or in combination with prebiotics (synbiotics) are now being explored for their therapeutic potential. Lactic acid bacteria used as probiotics exhibit a variety of biological actions including stimulation of the immune system, balancing of intestinal microbiota, potential reduction of inflammation and the prevention of allergies, hypertension and cancer [1]. Despite conferring several health benefits, the *in-vivo* survival and establishment of probiotic strains upon oral administration is elusive. Use of prebiotics such as fructooligosaccharides (FOS), galactooligosaccharides (GOS), and inulin can address some of these issues [2–4]. These carbohydrate-type prebiotics may however non-specifically encourage the growth of all gut organisms, including the non-probiotic pathogenic bacteria. Therefore, newer alternatives of non-carbohydrate origin need to be explored for stimulation of probiotic flora [5].

Green tea (*Camelia sinensis*), a rich source of polyphenols, is a widely consumed beverages in the world and is suggested to possess health promoting properties. Epigallocatechin gallate (EGCG) is the most abundant and the most active component of green tea leaves. It elicits various biological effects, including antimutagenicity and antitumorigenesis, free radical scavenging activity and antimicrobial activity against gut pathogens [6, 7]. Probiotic *L*. *plantarum* is reported to possess tannase activity [8, 9]; EGCG on the other hand is condensed tannin which yields phenolic acids, when hydrolyzed by tannase. In view of the tannase activity of *L*. *plantarum*, the possibility of using EGCG for enhancing growth of the former was explored in the present work.

Further, due to the limitation of bioavailability of EGCG and viability of *L*. *plantarum* during their transit through the harsh gut conditions, these agents were encapsulated in calcium alginate beads, resulting in a synbiotic formulation. Encapsulating probiotic with a natural polyphenolic molecule like EGCG for improved viability (of the former) and effectiveness of both the agents is the novelty of the system. The developed microbeads (microstructured synbox) will ensure a prolonged and continuous release of probiotic in the gut, allowing sufficient time for its adhesion and establishment on the gut mucosal wall. The idea of using natural molecules with their own set of suitable therapeutic activity in addition to supporting probiotic growth in a suitably designed pharmaceutical system is a relatively new concept. Application of the same was demonstrated earlier by us for proposed management of gastric ulcers [10]. It is noteworthy that EGCG with established antioxidant and anti-inflammatory effects is explored presently for its potential prebiotic effect for *L*. *plantarum*.

Thus, presently the developed synbiotic system was evaluated against oxidative stress/endotoxin mediated alcoholic liver disease (ALD). Alcohol induces damage by building up endotoxin mediated oxidative stress in the cellular constituents of the tissue. Catechins including EGCG have been demonstrated previously by us [11, 12] and others [13, 14] to attenuate alcoholic liver injury by creating an antioxidant- oxidant balance in the hepatic tissues. ALD is also accompanied by elevated intestinal permeability which can be abridged by probiotic administration. Recently, we have established the improved efficacy of *L*. *plantarum* [15] against ALD, when encapsulated within alginate beads. Similar enhancement for EGCG alone when encapsulated into alginate floating beads was also observed (data communicated). Presently we demonstrate the effects of combining these two agents (microstructured synbox) for a synergistic effect.

## Materials and Methods

### Agents

Natural polyphenol EGCG was provided as a gift sample by Y.S. Hara, Tea Solutions, Hara’s Office, Tokyo. Standard lactic acid bacteria (LAB)- *Lactobacillus plantarum* MTCC 2621, used as probiotic, was procured from Microbial Type Culture Collection (MTCC), Institute of Microbial Technology, Chandigarh (India).

### Construction of Microstructured Synbox

#### Effect of EGCG on the growth of *L*. *plantarum*

A dose dependent (10–200mg of EGCG) study was designed to observe the effect of EGCG on the growth of *L*. *plantarum* at 10^10^CFU/ml. Various concentrations of EGCG i.e 10mg/ml- 200mg/ml were added separately in the Lactobacillus MRS broth (Himedia, India) each containing 1% inoculum of the overnight *L*. *plantarum* 2621 culture. After 24 hours of incubation under anaerobic conditions at 37°C, the CFU were enumerated on MRS agar plates at 37°C following incubation for 48 hours.

In another set of experiment, various concentrations of inulin (10–200 μg) which is a widely used prebiotic was also evaluated for growth promoting effects on *L*. *plantarum*.

#### Preparation of probiotic- EGCG co-encapsulated alginate beads

*L*. *plantarum* and EGCG were encapsulated together in calcium alginate microbeads [16, 17] via extrusion technique. Briefly, *L*. *plantarum* (10^10^ CFU/ml) and EGCG (50 mg) were dispersed by stirring overnight in 1% sterile sodium alginate solution. Dispersion was added dropwise through a 26 G syringe needle into 1% sterile calcium chloride solution under stirring, and the beads formed spontaneously were left to harden in the former for 2 hours under continuous stirring. The beads were filtered out, using Whatman filter paper 1, and freeze dried for storage. The alginate loaded microparticles were named as AL.

### Characterization of probiotic-EGCG co-encapsulated alginate beads/microparticles

1. Size of microparticulate beads was determined using a stage micrometer and Olympus optical microscope.
2. Surface morphology of microparticles was examined using scanning electron microscope (SEM) (JSM- 6100, JEOL Ltd., Tokyo, Japan) housed in Central Instrumentation Laboratory/Sophisticated Analytical Instrument Facility of Panjab University, Chandigarh, India at 10 kV. The microparticles were mounted on metal grids using double-sided tape and coated with gold under vacuum.
3. Percent entrapment efficiency (EE %) of both the agents was determined using the following formulae:
  $$\text{EE of EGCG}\;(\%)=\frac{\text{EGCG entrapped in beads}}{\text{EGCG initially loaded in alginate mix}}\times 100$$
  $$\text{EE of}\;L.\;plantarum\;(\%)=\frac{\text{Log CFU entrapped in}\;100\;\text{g of beads}}{\text{Log CFU initially loaded in alginate mix}\;(\text{for}\;100\text{g beads})}\times 100$$
4. *In-vitro* release cum dissolution studies of EGCG and probiotic in alginate beads (equivalent to 10^10^ CFU/ml of probiotic and 50 mg of EGCG) were performed aseptically using the USP type II dissolution test apparatus at 100 rpm and 37± 0.5°C and 900 ml simulated intestinal fluid (SIF) (pH- 6.8) for 6 hours. 5ml aliquots of the medium were withdrawn at pre-determined time intervals and replaced with fresh dissolution media. The samples were analyzed for EGCG content spectrophotometrically at 270nm. For probiotic, spread plate method was employed.
5. Stability of EGCG in probiotic-EGCG combination beads was compared with that of free EGCG by dispersing suitable quantities of both in simulated intestinal fluid (pH-6.8) for 6 hours. At regular intervals, samples were withdrawn at similar time points and observed spectrophotometrically at 270nm and % stability was calculated.
6. The co-encapsulated beads were evaluated for their storage stability at 25°C in a stability chamber for 6 months. The bacterial count was enumerated by spread plate method on MRS agar plates at the start and at the end of the study
7. The stability of probiotic in probiotic- EGCG alginate beads was evaluated in the presence of bile salts, by suspending the beads, in 0.3% bile salt solutions (sodium taurocholate and sodium glycolate) for 4 hours.
  $$\text{Survivability of}\;L.\;plantrum\;\text{in Bile Salts}\;(\%)=\frac{{\text{Log}}_{10}\;\text{CFU at}\;4\;\text{hours}}{{\text{Log}}_{10}\;\text{CFU at}\;0\;\text{hour}}$$
8. The probiotic- EGCG alginate beads and free probiotic were incubated in simulated gastric fluid (SGF) (pH-1.2) for 4 hours and sequentially in SIF (pH-6.8) for 2 hrs and the CFU remaining in each case were determined to establish viability of probiotic in either case.

$$\text{Survivability of}\;L.\;plantarum\;\text{in SGF}\;\&\;\text{SIF}\;(\%)=\frac{{\text{Log}}_{10}\;\text{CFU at}\;6\;\text{hours}}{{\text{Log}}_{10}\;\text{CFU at}\;0\;\text{hour}}\times 100$$

### *In-vivo* Studies

#### Ethics statement

The experiment protocols were approved by the Institutional Animal Ethics Committee (Approval ID- IAEC/282/ dated– 30/8/2012) of Panjab University, Chandigarh, India and performed in accordance with the guidelines of Committee for the Purpose of Control and Supervision of Experiments on Animals (CPCSEA), Government of India, on animal experimentation. All efforts were made to minimize the suffering of animals.

#### Animals

Female wistar rats (200–250 g) were procured from Central Animal House, Panjab University, Chandigarh (India). The animals were housed under standard laboratory conditions, maintained under normal light: dark cycle and had free access to food (Ashirwad Industries Pvt. Ltd., Punjab, India) and water.

### Establishment of ALD in Wistar rats

#### Alcohol dosing

Rats were administered 10g/kg of body weight/day of 35% (v/v) ethanol (obtained from Brampton, Ontario) by oral gavage in double distilled water for two weeks. Thereafter, the dose was increased to 14g/kg of body weight/day and was continued for 10 weeks by oral gavage [11].

#### Combination bead dosing

The *L*. *plantarum* and EGCG combination beads, as prepared above, were dispensed in 1% carboxymethyl cellulose and were administered to rats for 8 weeks through oral gavage.

#### Experimental Design

**Fig 1:**
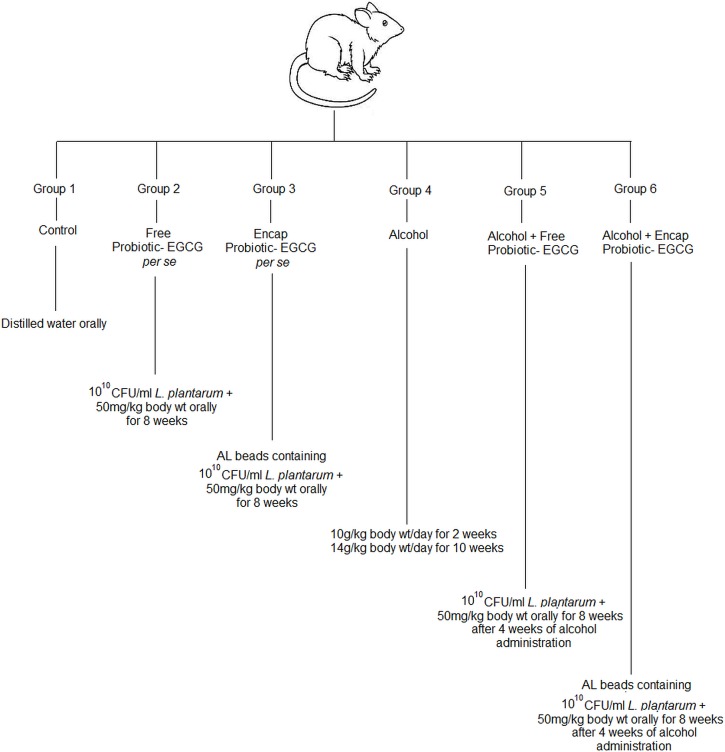
Diagrammatic representation of various treatment groups made for *in-vivo* studies.

After an acclimatizing period, rats were randomly divided into six groups, each comprising of 8–10 rats (Fig 1). Rats were administered alcohol as described above and the dose of alcohol group was selected on the basis of the previous study [11, 15]. Similarly, treatment using *L*. *plantarum* and EGCG microstructured synbox was carried on for 8 weeks. At the end of the experimental period (after 12 weeks), the rats were sacrificed by cervical dislocation. Livers were removed quickly, rinsed in cold phosphate buffer saline (0.05 M, pH 7.4) and stored at -62°C till further use.

#### Measurement of blood alcohol

After 12 weeks of alcohol administration, blood was taken from the tail vein 1.5 h and 2.5 h after the last dose. Blood alcohol levels (BAL) were measured using the alcohol dehydrogenase kit procured from Sigma Chemical Co., U.S.A.

#### Plasma endotoxin assay

Endotoxin level in the plasma samples was measured using Toxin Sensor Chromogenic LAL Endotoxin Assay Kit (Hycult Biotech) as described by us earlier [11].

#### Assessment of liver function

Alanine aminotransferase (ALT) and aspartate aminotransferase (AST) enzyme activities in serum were determined using ERBA test kits (ERBA Diagnostics, Mannheim, Germany). Alkaline phosphatase (ALP) was estimated using Enzopak Diagnostic kit (Reckon Diagnostics, India).

#### Tissue architecture studies

Liver tissues removed aseptically from the animals were cut into small pieces and fixed in 10% buffered formalin. Samples were processed, stained with hematoxylin-eosin and examined under the light microscope.

#### Assay for potential antioxidant capacity/ Total antioxidant capacity test

Commercially available (DTAC-100, Bioassay Systems) total antioxidant capacity kit was used as per the manufacturer’s instructions.

#### Determination of intestinal permeability

Method [18] is included in supplementary information (S1 Text). Briefly, two non-metabolizable sugars, lactulose (Himedia, India) and mannitol (Himedia, India) were administered orally to overnight fasted animals of various groups, and their concentrations were measured in the serum of these animals using HPTLC, one hour post administration.

#### Assay for NF-kB/p50, TNF-α and IL12/p40 subunit

Assay for NF-kB/p50 subunit in the nuclear extracts and TNF- *α* in the liver homogenates, was performed using commercially available Transcription Factor Assay kit (Upstate Biotechnology, NY, USA) and cytokine assay kit (R&D Systems, USA), respectively, according to the manufacturer’s instructions [11].

For IL12/p40 subunit estimations a double antibody sandwich ELISA was performed using manufacturer’s instructions of the commercially available kit (Qaybee-bio, China).

#### Transcription studies of signalling molecules

Liver tissue of the rats subjected to the indicated treatments were harvested and immediately preserved in RNA*later* (Ambion, CA, USA) at -80°C till processing. Total RNA was isolated using the RNeasy Mini Kit (Qiagen) according to the manufacturer’s instructions and was, then, reverse transcribed by using oligo-dT primer and first strand cDNA synthesis kit (Fermentas). Obtained cDNA was subjected to PCR using TLR4, CD14, MD2, MyD88, COX-2 and Glyceraldehyde-3-phosphate dehydrogenase (GAPDH)-specific primers (Sigma Aldrich Chemicals, Banglalore, India). GAPDH mRNA was used as an internal control. Densitometry of PCR product to determine relative mRNA expression was performed by Gel Doc Multi-Analyst (BioRad USA).

#### Micronucleus analysis

Micronuclei analysis was done by the method of Schmid [19] which is elaborated in the supplementary information (S2 Text). Minimum of 100 cells were counted per sample for the presence of micronuclei using light microscope at 45x.

#### Statistical analysis

The data are expressed as mean ± standard deviation. Statistical significance between various groups was evaluated using one way analysis of variance (ANOVA) followed by Tukey’s multiple comparison tests. The statistical analysis was done using the Graphpad Prism 6.00 for Windows (Graphpad software, California, USA). In all data analysis, p-values of 0.05 or less (p˂0.05) were considered significant.

## Results

### Effect of EGCG on the growth of *L*. *plantarum*

**Fig 2:**
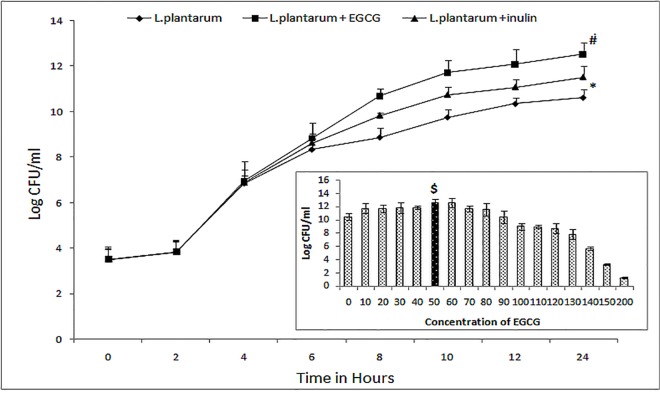
Comparative effect of EGCG and inulin on the growth of *L*. *plantarum*. Inset-Enhancing effect of various doses of EGCG on the growth of *L*. *plantarum*. Values are expressed as mean ± standard deviation of three individual values. $, 50mg dose displays most significant effect (P>0.05), *, P <0.05 versus log_10_ CFU of *L*. *plantarum* after 24 hour in the absence of EGCG and inulin (control); #, P <0.05 versus log_10_ CFU of *L*. *plantarum* after 24 hour in the presence of inulin.

Growth of the probiotic was found to increase with increasing concentrations of EGCG upto 60mg, subsequent to which, a hormesis effect was observed. Growth enhancing effect of EGCG for *L*. *plantarum* was significantly (p ≤0.05) more (10.55±0.78 to 12.8 ±0.5 log_10_ units) than that induced by inulin, the most commonly used prebiotic (10.55± 0.78 to 11.4±0.38 log_10_ units) at corresponding concentrations (Fig 2).

### Co-microencapsulation of probiotic-EGCG and its characterisation

#### 1) Size of microparticles and Scanning Electron Microscopy (SEM) images

**Fig 3:**
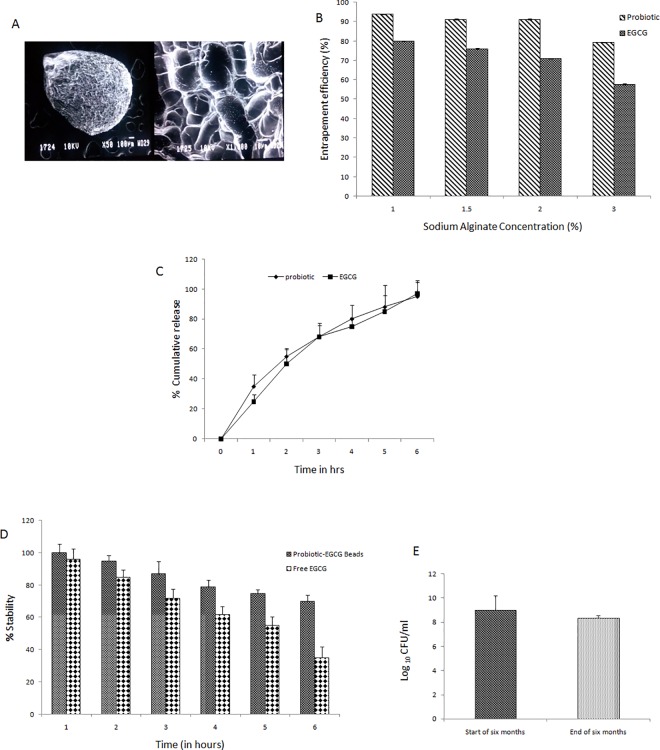
Characterization of formulated beads on the basis of entrapment efficiency, SEM, stability of EGCG in alkaline pH, shelf life of probiotic for 6 months. A) Scanning electron micrographs (a) Probiotic-EGCG beads at 50X (b) Probiotic-EGCG beads at 1000X. B) Drug entrapment efficiency of probiotic and EGCG co-encapsulated in alginate beads (n = 6). C) *In-vitro* release of entrapped probiotic and EGCG from probiotic-EGCG beads upto 6 hours. D) Stability of EGCG in probiotic- EGCG alginate beads as compared to free EGCG in alkaline pH after 6 hours.(n = 6) * represents p<0.05 versus free EGCG. E) Shelf-life of probiotic for 6 months. Values are represented as ± SD. * represents p<0.05 versus probiotic at the start of 6 months.

Size of probiotic-EGCG beads significantly increased to 75.4±7.8μm as compared to the beads incorporating only probiotic as described earlier [15]. SEM micrographs show smooth surface and presence of probiotic rods in the beads (Fig 3A).

#### 2) Percentage entrapment efficiency of combination beads

The entrapment efficiency of both probiotic and EGCG decreased with an increase in the concentration of sodium alginate. The maximum EE of 94.00±0.13% for probiotic and 80.00±0.21% for EGCG was observed with 1% sodium alginate. Minimum entrapment was observed with the highest tried concentration (3%) of sodium alginate (79.40±0.13% for probiotic and 57.74±0.34% for EGCG) (Fig 3B). EE of probiotic when entrapped alone was found to be 80% [15] but upon co-encapsulation with EGCG, it increased considerably to 94%.

#### 3) *In-vitro* release studies

Significant quantities of probiotic (95%) and EGCG (97%) were released from the probiotic-EGCG beads within 6 hours (Fig 3C).

#### 4) Stability of EGCG at intestinal pH

EGCG entrapped in probiotic-EGCG combination was significantly more stable than free EGCG under alkaline conditions (representing pH of the intestine) ensuring better protection (Fig 3D).

#### 5) Shelf life of probiotic-EGCG beads at room temperature

The probiotic bacterial counts observed at the end of 6 months of storage were the same as at the start of the study (log_10_ 8.98 to log_10_ 8.36 CFU/ml; p ≤ 0.05) (Fig 3E). Therefore, probiotic entrapped in probiotic-EGCG combination beads was quite stable at the end of six months.

#### 6) Survivability of probiotic entrapped in probiotic- EGCG beads in SGF, SIF and Bile salts

The probiotic *L*. *plantarum* was able to strive significantly (p<0.05) better through the harsh simulated gut environment when encapsulated in the beads. The percentage survivability of encapsulated cells was 72.2% at the end of the study. Similar results were obtained on exposure to bile salts (S1 Table).

### In-vivo studies

#### Blood alcohol levels

After 12 weeks of regular alcohol administration, the blood alcohol levels (BAL) were significantly increased upto 243.2 mg/dL and 198.6 mg/dL at 1.5 h and 2.5 h, respectively, after administration of last dose. The levels were however significantly (p≤0.05) lowered to 197.4 and 154.1 mg/dL, respectively, in the probiotic- EGCG beads supplemented group after 1.5 and 2.5 h of the last administered dose.

#### Plasma endotoxin levels

The plasma endotoxin levels in the control rats were observed to be 0.16 EU/ml. Chronic alcohol consumption considerably increased the endotoxin levels to 0.54 EU/ml. However, on co-supplementing alcohol administration with probiotic- EGCG beads, the elevated endotoxin levels were significantly reduced to 0.11 EU/ml (p<0.001).

#### Liver function tests

Alcohol caused a marked rise in the serum levels of ALT and ALP while there was no significant change in AST levels. Activities of liver enzymes dropped significantly on co-supplementation with encapsulated probiotic -EGCG (S2 Table).

#### Hepatic tissue architecture

**Fig 4:**
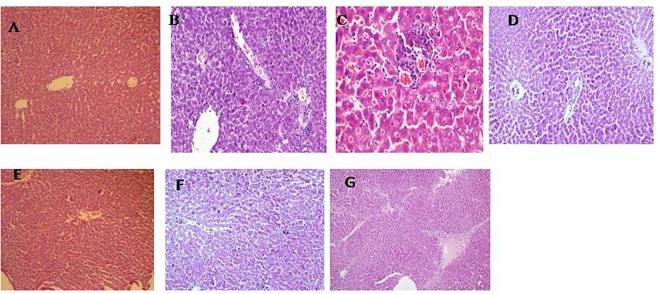
Representative photomicrographs of hematoxylin-eosin stained rat liver sections. (A) Normal rat liver (100x); B,C) Liver section from rat administered 10-14g/kg body weight of 35% alcohol orally for 12 weeks showing vacuolar degeneration, microvesicular fatty change, focal collection of lymphocytes and vascular congestion (200X, 400X respectively); D) Photomicrograph of alcohol administered co-supplemented with free probiotic-EGCG group showing normal histology (100X); E) Photomicrograph of alcohol administered co-supplemented with encapsulated probiotic—EGCG group showing normal histology (100X); F) Photomicrograph of Free probiotic-EGCG *per se* group showing normal histology (100X); G) Encapsulated probiotic +EGCG *per se* group showing normal histology (100X).

Liver of alcohol administration group showed vacuolar degeneration, micro- and macrofollicular fatty changes and focal collection of lymphocytes in the liver. Portal tract inflammation (portal triaditis) was also observed (Fig 4B and 4C). In contrast, the histological examination revealed normal liver with no fatty change in both the free probiotic- EGCG and the co-encapsulated group (Fig 4D and 4E). The livers of *per se* groups (Fig 4F and 4G) were also observed to be normal (Fig 4A).

#### Potential antioxidant levels

**Fig 5:**
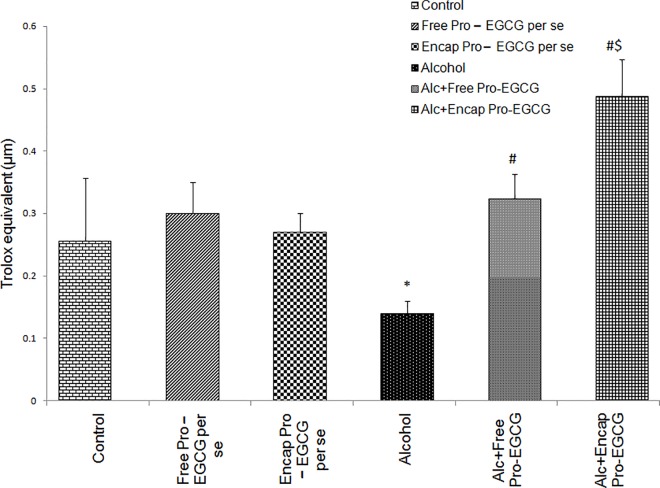
Effect of probiotic- EGCG combination (free and encapsulated) on PAO levels, *p<0.05 vs. control, ^#^p<0.05 vs. alcohol group, $p<0.05 vs. alcohol + free probiotic-EGCG group.

The potential antioxidant (PAO) capacity test, measured as trolox equivalent (μM) was performed in the rat serum (Fig 5). Values observed in case of alcoholic rats were significantly lower than control and treated rats. The entrapped probiotic-EGCG treated group showed values even greater than the control animals (p ≤ 0.05). P*er se* groups were similar to the control group. Since the PAO levels of animals treated with probiotic- EGCG co-encapsulated beads was significantly increased, hence, individual antioxidants (SOD, catalase and glutathione reductase) were also studied in this group (data not shown). Levels for each of these moeities were significantly higher than the control and the ALD group.

#### Assessment of intestinal permeability

**Fig 6:**
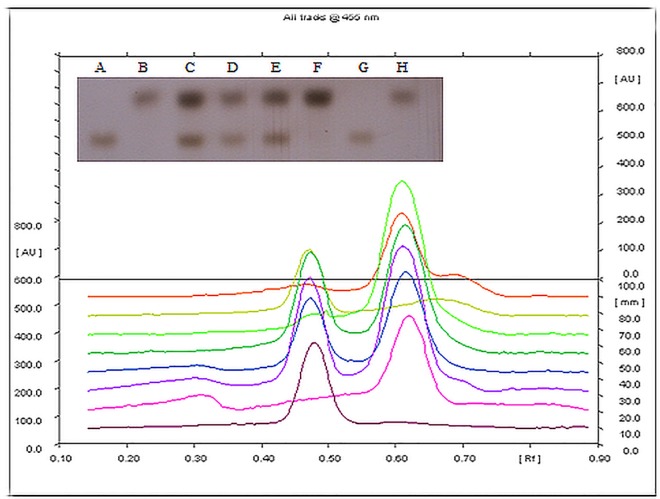
HPTLC Chromatogram (inset) and chromatograph showing the differential sugars i.e. lactulose and mannitol released as a marker of intestinal permeability. Inset- A,G–lactulose standard; B,H—mannitol standard; C- Alcohol group showing the presence of both the sugars in the serum due to increased permeability; D- Alcohol + Encapsulated probiotic-EGCG group showing only mannitol, no disruption of gut permeability; E- Alcohol fed; F- Alcohol + Free probiotic-EGCG group showing only mannitol, confirming intact gut permeability

The compromised intestinal permeability marks the onset of ALD as it allows the releases of endotoxins into the serum. Increased intestinal permeability on chronic alcohol administration was confirmed by the presence of bands of both lactulose (disaccharide) and mannitol (monosaccharide) in serum, indicating their transport across the gut wall. Treatment with entrapped probiotic-EGCG reduced the permeability as only mannitol (monosaccharide) band was observed on the plate. The EGCG beads, on the other hand had no effect on the intestinal permeability which was elevated by alcohol administration (Fig 6 inset). Rf of mannitol was recorded as 0.49 whereas the Rf of lactulose was 0.61.

### Molecular mechanisms of protection

#### Assay for NF-κB/p50, TNF-α and IL-12/40 subunit

**Fig 7:**
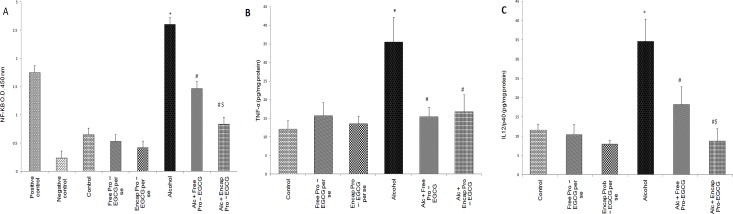
**Effect of probiotic- EGCG combination (free and encapsulated)** on A) NF- B/p50, B) TNF-α levels, and C) IL-12/p40 levels in alcohol administered rats with and without treatment. Values are expressed as mean± S.D. of eight different observations. *p<0.05 vs. control, ^#^p<0.05 vs. alcohol group, $p<0.05 vs. alc+ free pro-EGCG group.

The alcohol treated rats showed a marked escalation of TNF-α and IL-12/p40 subunit values in comparison to those exhibited in the control group. On co-supplementing with probiotic- EGCG beads, the enhanced (p<0.05) levels were attenuated to normal values (Fig 7B and 7C). NF-ĸB/p-50 was also inactivated by treatment with both the free and the encapsulated probiotic- EGCG, with the effect (p<0.05) being more pronounced for the latter, which elaborated a complete reversal of NF- κB/p50 activation manifested by alcohol treatment (Fig 7A).

#### mRNA expression of signalling molecules

**Fig 8:**
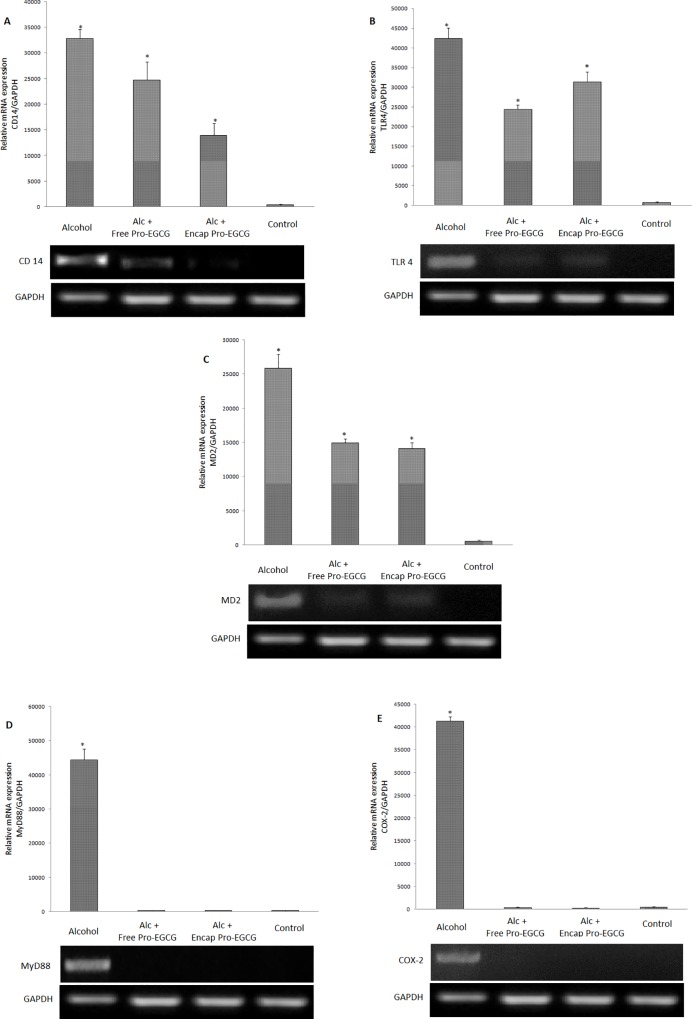
RT-PCR analysis of liver TLR4, CD14, MD2, MyD88 COX-2 mRNA expressions. Alc- alcohol group; Alc+ FPE- Alcohol+ free probiotic- EGCG group, Alc + EPE- Alcohol+ co-encapsulated probiotic-EGCG group. *p<0.05 vs. control.

An upregulated mRNA expression was observed in all the signaling molecules i.e. TLR4, CD14, MD2, MyD88 and COX-2 in the alcohol group. However, co-supplementing the alcohol fed rats with probiotic-EGCG beads led to their complete downregulation (Fig 8).

#### Micronuclei analysis

**Fig 9:**
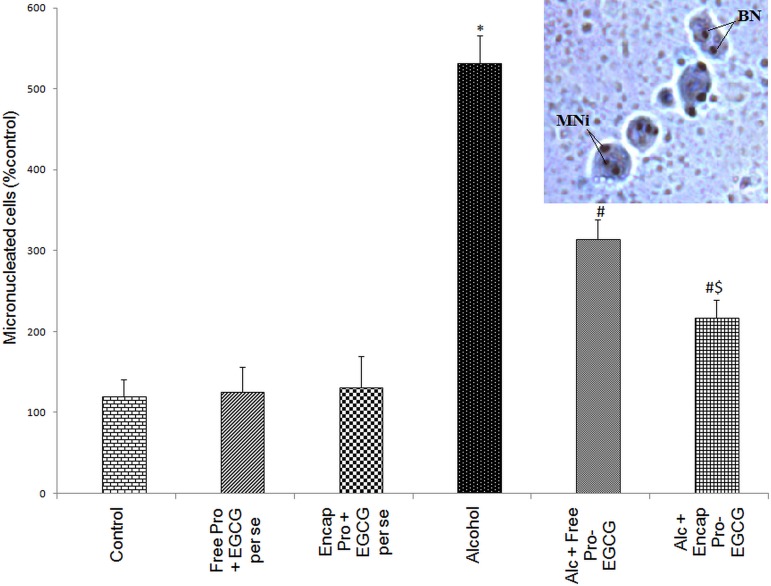
Micronuclei analysis in the hepatocytes of alcohol-fed rats. Effect of co-enapsulated Probiotic-EGCG on the extent of micronuclei formation in hepatocytes of alcohol administered rats. Values are expressed as percentage of micronucleated cells. *p<0.001 vs. control, ^#^p<0.01 vs. control, ^#$^p<0.05 vs. control; **Inset**- Dividing cells showing binuclei (BN) and micronuclei (MN) in hepatocytes of alcohol-fed rats.

A significant increase in the micronucleated cell score was observed after alcohol abuse. Fig 9. represents the binucleated and micronucleated cells in alcohol induced liver tissue. However, co-supplementation with the co-encapsulated probiotic-EGCG to alcohol-fed rats resulted in a significant decrease in micronuclei.

## Discussion

Proper management of alcoholic liver disease, although an oldest form of liver disease, is still lacking due to safety issues associated with use of indicated treatment options. Though, alternative herbal therapies [20] are suggested for its treatment but the evidence based clinical studies to this fact are limited. Suitability of probiotic supplementation is also indicated for control of ALD [21–24], howsoever their survival and establishment in the intestinal mucosa, following transit through the harsh gut conditions is must for appropriate treatment outcomes. To address the issue of efficiently delivering the probiotic to the gut mucosa, we have reported earlier that microencapsulation protects probiotics from adverse gut conditions as was shown for effective management of ALD [15] and gastric ulcers [10]. It is important to note that survivability and metabolic activity of the encapsulated probiotic also needs to be maintained throughout the production process and following administration. Use of prebiotics for enhanced survival of probiotic bacteria has also been widely suggested, as these agents serve as the energy source of probiotics [25, 26].

In the present study a rodent model of ALD was used to investigate the effect of suitably designed “microstructured synbox” co-encapsulating *L*. *plantarum* and EGCG, taken as a prebiotic source, in calcium alginate beads. The cellular and molecular parameters were assessed to delineate the possible pathways involved in protection offered by this dual therapy.

EGCG, a condensed tannin, highly endorsed for its anti-oxidative and anti-inflammatory activity was evaluated for its prebiotic potential in the present study. EGCG was selected for its phenolic nature and the fact that phenol decarboxylase and inducible acid phenol reductase activities possessed by *L*. *plantarum* endow it with the capacity to metabolize phenolic acids [8,9,27]. This will make EGCG a highly specific growth promoter, while inulin a general polysaccharide may equally support the growth of gut associated pathogenic bacteria. EGCG demonstrated significant and better prebiotic effect for *L*. *plantarum*, in comparison to inulin, the well established prebiotic [28]. Howsoever, the growth promoting effect of EGCG was observed only upto 60 mg beyond which hormesis [29] might set in; that may be due to the accumulation of toxic metabolites which exacerbate stress related responses including cell detrimental pH alterations [30]. Hormesis is defined as adaptive response of cells and organisms to intermittent stress [29].

Sodium alginate showed optimum entrapment efficiency to co-encapuslate EGCG and *L*. *plantarum* at 1% concentration. Higher concentrations increase the viscosity of the solution reducing its mobility during stirring and hence a reduced entrapment was observed. Presence of EGCG conferred (i) improved probiotic survivability against harsh GIT conditions in simulated studies (acidic pH and presence of bile salts), and (ii) better entrapment (94%) of probiotic in the ‘synbox’ in contrast to when probiotic was used alone (80% entrapment). EGCG being a large molecule (140 kDa), probably plugs the alginate mesh both against the entry of acidic, enzymatic and alkaline contents of gut and leakiness of the entrapped *L*. *plantarum*. Size of the beads increased significantly when both EGCG and probiotic were incorporated as compared to when these agents were entrapped individually. These results are in concordance with an earlier study wherein size of microparticles varied with the type and content of probiotic and prebiotic used [31]. SEM images of the ‘microstructured synbox’ revealed a smooth surface with the observance of *L*. *plantarum* on the surface.

In order to predict the performance of the ‘synbox’, the beads were tested *in-vitro* (under simulated conditions) for their potential to preserve the viability of entrapped probiotic under adverse conditions encountered in the stomach (extreme pH conditions) and intestine (microaerophilic conditions, bile salts). The co-encapsulated beads showed significantly better results (in terms of survival rate in simulated condition, *in-vitro* release and shelf-life) than those exhibited by probiotic [15] and EGCG beads individually (data communicated elsewhere). And and Kaliaspathy [32] reported similar results wherein the incorporation of Himaize starch increased the survival rate of *L*. *acidophilus*.

In the present study, the observed BAL in rats confirms the appropriate alcohol consumption and its break down in the liver to generate potentially dangerous by-products that contribute to alcohol induced liver damage. It may be said that the excessive alcohol consumption led to endotoxemia (elevated plasma endotoxemia levels in alcohol administered rats) which further attributed to the leaky gut syndrome as observed by the increased epithelial and paracellular transport of both lactulose (a dissacharide) and mannitol (a monosaccharide) in the alcoholic rats. Similar clinical conditions are detected in patients with alcoholic hepatitis. ‘Microstructured synbox’ treated group gave a direct indication of restoration of tight junctions as evidenced by presence of only mannitol in the in the serum of these animals.

The compromised gut permeability following high intake of alcoholic beverages also promotes systemic passage of carcinogenic substances leading to decreased cell metabolism and cellular immunity followed by DNA damage and cell death [33]. We presently report an increased formation of micronuclei (MNi) (which originates from acentric chromosome fragments or whole chromosomes that are not included in the main daughter nuclei during nuclear division) in the hepatocytes of ALD rats taken as an indicator of genotoxic response to carcinogenic agents [34, 35]. The MNi frequency was reduced in the ‘microstructured synbox’ treated group.

Liver inflammation and damage was indicated by the liver function tests. The elevated levels of hepatic markers, (ALT and ALP) were restored after administration of combination beads (supplementary data). The liver histoarchitecture was restored in the alcohol fed and combination beads treated group.

Another factor that controls the pathophysiology of ALD is generation of ROS due to the activation of kupffer cells in liver that causes oxidative stress. The PAO assay performed in the present study indicated increased antioxidant levels (of SOD, catalase and GSH) in treatment group.

Evidence suggests that endotoxin mediated overwhelmed (redox imbalance) system leads to activation of stress sensitive signaling pathways such as induction of NF-kB. NF-kB dependent gene expression in kupffer cells contribute to alcohol induced liver injury [21, 36–39] through inflammatory mediators including TNF-α. Significant inhibition of activation of NF-kB by co-encapsulated beads, might have presently, suppressed the liver injury. This is consistent with reports where green tea polyphenols have been shown to inhibit NF-KB activation in ischaemia reperfusion liver injury and streptozotocin induced diabetic rats [40]. This may further down regulate TNF-α [12], as also observed presently, coupled with the restoration of antioxidative status.

Though, TNF-α is a promising target for various therapeutic diseases, yet certain side effects are now associated with its use in immunocompromised patients. Thus newer therapeutic targets [41] viz. IL-12/p 40 which when downregulated, blocks IL-12, IL-23 and TNF-α, are being explored. Animals treated presently with co-encapsulated beads brought down the levels of not only TNF-α but also IL-12/p 40 subunit.

In continuation, diminished expression of TLR4, MD2, CD14, MyD88 and COX-2 was recorded as compared to their strong expression in case of alcohol administered rats. This lower gene expression of the signalling molecules after treatment with ‘microstructured synbox’ indicated the blockade in the binding of LPS to TLR4, therefore, downregulaing the other associated effector molecules. It is known that endotoxin binds specifically to TLR4, a trans-membrane protein. TLR4 in association with MD2 recognizes the LPS/ CD14 complex. Thus, CD14, TLR4 and MD2 are the major intrinsic components of receptor complex which play an important role in signal transduction of LPS. The TLR4/MD2 complex further activates a signaling pathway via the recruitment of adaptor proteins including MyD88 and cvclooxygenase-2 (COX-2), an inducible enzyme of macrophages catalysing the conversion of arachidonic acid to prostaglandins which are potent inflammatory mediators causing hepatic injury. Our findings revealed that combination beads significantly inhibited the expression of these genes and transduction of signals at the membrane level disrupting the intracellular activation. We observed similar results in an earlier study where catechin acted as a chain breaking inhibitor of the signalling molecules involved in LPS signalling [11]. These results are also in accordance with an earlier study where *Lactobacillus amylovorus* has been found to inhibit TLR4 signalling triggered by enterotoxigenic *E*. *coli* in Caco-2 cell lines and pig explants [42]. Present study thus provides an insight on the enhanced efficacy of developed EGCG-probiotic co-encapsulated synbox beads in suppressing an array of molecules operative in the pathogenesis of ALD.

The above mentioned observations indicated that alcohol gavage caused significant endotoxaemia coupled with decreased activities of hepatic antioxidants and increased frequency of micronuclei generation in rats. Treatment with EGCG-probiotic coencapsulated beads attenuated levels of endotoxin, increased hepatic antioxidants along with decrease in the number of micronuclei and amelioration of disruptive histoarchitecture. This may be due to the antioxidative properties of EGCG and the probiotic. Flavonoids are known to localize near the membrane surface, trapping directly any free radicals generated in lipid environment or in the aqueous phase, while probiotics reduce the oxidative stress due to their ability to improve gut barrier function by maintaining the intestinal permeability [21]. Combination beads thus showed a multifactorial effect against increased endotoxin levels and genotoxicity, as well as disrupted histoarchitecture and antioxidative status.

## Conclusions

Our findings suggesting sequential inhibition of signal transduction without incurring prohibitive toxicity or loss of innate immunity, may be of importance in designing strategies like the presently used combination of *L*. *plantarum* with EGCG in a ‘synbox’ for management of this highly prevalent and significant clinical manifestation. Use of these agents, alone or in conjunction with conventionally prescribed drugs may lower the dose and hence associated side-effects of latter, besides conferring significant complementary health benefits. These results need to be confirmed in humans in order to validate the applicability of the prepared formulation. Thus, it is important to realise that full potential of such folkloric natural remedies can be exploited only if the basic biology is combined with the modern day technology to result in effective therapies.

## Supporting Information

S1 Table Log_10_ CFU of *L*. *plantarum* entrapped in probiotic- EGCG beads in SGF, SIF and Bile salts. (DOCX)

S2 Table Effect of free probiotic and encapsulated probiotic-EGCG on hepatic markers in the serum of control and alcohol-administered rats. (DOCX)

S1 Text Micronuclei analysis method. (DOCX)

S2 Text Measurement of intestinal permeability. (DOCX)

## References

[1] Park JH, Kim Y, Kim SH. Green tea extract (*Camellia sinensis*) fermented by *Lactobacillus fermentum* attenuates alcohol-induced liver damage. Biosci Biotechnol Biochem. 2012; 23: 2294–2230. 10.1271/bbb.120598 23221715

[2] Kolida S, Tuohy K, Gibson GR. Prebiotic effects of inulin and oligofructose. Bri J Nutri. 2002; 87: S193–197. 10.1079/BJNBJN/2002537 12088518

[3] Babu G, Nithyalakshmi V. Influence of prebiotic composition on probiotic survivability in calcium alginate coated symbiotic microcapsules at thermal incubation. Agriculture J. 2011; 6: 231–236.

[4] Rishi P, Mavi SK, Bharrhan S, Shukla G, Tewari R. Protective efficacy of probiotic alone or in conjunction with a prebiotic in *Salmonella*-induced liver damage. FEMS Microbiol Ecol. 2009; 69: 222–230. 10.1111/j.1574-6941.2009.00703.x 19496820

[5] Vodnar DC, Socaciu C. Green tea increases the survival yield of Bifidobacteria in simulated gastrointestinal environment and during refrigerated conditions. Chem Central J. 2012; 6: 61. 10.1186/1752-153X-6-61 PMC3408365 22727242

[6] Si W, Gonga J, Tsaoa R, Kalab M, Yang R, Yin Y. Bioassay-guided purification and identification of antimicrobial components in Chinese green tea extract. J Chromatography. 2006;1125: 204–210 10.1016/j.chroma.2006.05.061 16797571

[7] Du GJ, Zhang Z, Wen XD, Yu C, Calway T, Yuan CS et al Epigallocatechin Gallate (EGCG) is the most effective cancer chemopreventive polyphenol in green tea. Nutrients. 2012; 4: 1679–1691. 10.3390/nu4111679 23201840 PMC3509513

[8] Osawa R, Kuroiso K, Goto S, Shimizu A. Isolation of tannin-degrading Lactobacilli from humans and fermented foods. Appl Environ Microbiol. 2000; 66: 3093–3097. 10877812 10.1128/aem.66.7.3093-3097.2000 PMC92117

[9] Vaquero I, Marcobal A, Muñoz R. Tannase activity by lactic acid bacteria isolated from grape must and wine. Int J Food Microbiol. 2004; 96: 199–204. 10.1016/j.ijfoodmicro.2004.04.004 15364474

[10] Singh PK, Kaur IP. Synbiotic (probiotic and ginger extract) loaded floating beads: a novel therapeutic option in an experimental paradigm of gastric ulcer. J Pharm Pharmacol. 2012; 64: 207–217. 10.1111/j.2042-7158.2011.01397.x 22221096

[11] Bharrhan S, Koul A, Chopra K, Rishi P. Catechin suppresses an array of signalling molecules and modulates alcohol-induced endotoxin mediated liver injury in a rat model. Plos One. 2011; 6: e2063. 10.1371/journal.pone.0020635 PMC3108820 21673994

[12] Bharrhan S, Chopra K, Arora SK, Toor JS, Rishi P. Down-regulation of NF-{kappa}B signalling by polyphenolic compounds prevents endotoxin-induced liver injury in a rat model. Innate Immun. 2011; 18: 70–79. 10.1177/1753425910393369 21239456

[13] Yuan G, Gong Z, Zhou X, Zhangq P, Sun X, Li X. Epigallocatechin-3-Gallate ameliorates alcohol-induced liver injury in rats. Int J Mol Sci. 2006; 7: 204–219.

[14] Kaviarasan S, Sundarapandiyan R, Anuradha CV. Epigallocatechin Gallate, a green tea phytochemical, attenuates alcohol-induced hepatic protein and lipid damage. Toxicol Mechanisms Methods. 2008; 18: 645–652. 10.1080/15376510701884985 20020850

[15] Arora S, Kaur IP, Chopra K, Rishi P. Efficiency of double layered microencapsulated probiotic to modulate pro-inflammatory molecular markers for the management of alcoholic liver disease. Mediators Inflammat. 2014: 1–11. 10.1155/2014/715130 PMC4055561 24966470

[16] Krasaekoopt W, Bhandari B, Deeth H. Evaluation of encapsulation techniques of probiotics for yoghurt. Int Dairy J. 2003; 13: 3–13.

[17] Krasaekoopt W, Bhandari B, Deeth H. The influence of coating materials on some properties of alginate beads and survivability of microencapsulated probiotic bacteria. Int Dairy J. 2004; 14: 737–743.

[18] Noth R, Lange-Gumfeld J, Stuber E, Kruse ML, Ellrichmann M, Häsler R, et al Increased intestinal permeability and tight junction disruption by altered expression and localization of acculin in a murine graft versus host disease model. BMC Gastroenterol. 2011; 11: 109, 10.1186/1471-230X-11-109 21977944 PMC3198696

[19] Schmid S. An evaluation of the micronuclei test using triethylenemelamine, trimethylphosphate and niridazole. Mutat Res. 1975; 28: 101–106. 1095914 10.1016/0027-5107(75)90319-x

[20] Kim MS, Ong M, Qui X. Optimal management for alcoholic liver disease: Conventional medications, natural therapy or combination? World J Gastroenterol. 2016; 22: 8–23. 10.3748/wjg.v22.i1.8 26755857 PMC4698510

[21] Forsyth CB, Farhadi A, Jakate SM, Tang Y, Shaikh M, Keshavarzian A. *Lactobacillus* GG treatment ameliorates alcohol-induced intestinal oxidative stress, gut leakiness, and liver injury in a rat model of alcoholic steatohepatitis. Alcohol. 2009; 43: 163–172. 10.1016/j.alcohol.2008.12.009 19251117 PMC2675276

[22] Chang B, Sang L, Wang Y, Tong J, Zhang D, Wang B. The protective effect of VSL#3 on intestinal permeability in a rat model of alcoholic intestinal injury. BMC Gastroenterol. 2013; 13: 1–8 24138544 10.1186/1471-230X-13-151 PMC4016537

[23] Li F, Duan K, Wang C, McClain C, Feng W. Probiotics and alcoholic liver disease: Treatment and potential mechanisms. Gastroenterol Res Pract. 2016; Article ID 5491465, 11 pages. 10.1155/2016/5491465 PMC4709639 26839540

[24] Barone R, Rappa F, Macaluso F, Caruso Bavisotto C, Sangiorgi C, Di Paola G, et al Alcoholic liver disease: A mouse model reveals protection by *Lactobacillus fermentum*. Clin Transl Gastroenterol. 2016; 7:e138 10.1038/ctg.2015.66 26795070 PMC4737872

[25] Yan AW, Fouts DE, Brandl J, Stärkel P, Torralba Schott E. Enteric dysbiosis associated with a mouse model of alcoholic liver disease. Hepatology. 2011; 53:96–105. 10.1002/hep.24018 21254165 PMC3059122

[26] Li P, Burr GS, Gatlin DM, Hume ME, Patnaik S, Castille FL, et al Dietary supplementation of short-chain fructooligosaccharide influences gastrointestinal microbiota composition and immunity characteristics of Pacific white shrimp, *Litopenaeus vannamei*, cultured in a recirculating system. J Nutr. 2007; 137:2763–2768 18029496 10.1093/jn/137.12.2763

[27] Barthelmebs L, Divies C, Cavin JF. Molecular characterization of the phenolic acid metabolism in the lactic acid bacteria *Lactobacillus plantarum*. Le Lait. 2001; 81: 161–171.

[28] Kolida S, Tuohy K, Gibson GR. Prebiotic effects of inulin and oligofructose. Br J Nutr. 2002; 87: S193–S197 10.1079/BJNBJN/2002537 12088518

[29] Mattson MP, Hormensis defined. Ageing Res Rev. 2008; 7:1–7. 10.1016/j.arr.2007.08.007 18162444 PMC2248601

[30] Axling U, Olsson C, Xu J, Fernandez C, Larsson S, Ström K et al Green tea powder and *Lactobacillus plantarum* affect gut microbiota, lipid metabolism and inflammation in high-fat fed C57BL/6J mice. Nutr Metab. 2012; 9:105. 10.1186/1743-7075-9-105 PMC3538623 23181558

[31] Chávarri M, Marañón I, Ares R, Ibáñez FC, Marzo F, del Carmen Villaran M. Microencapsulation of a probiotic and prebiotic in alginate-chitosan capsules improves survival in simulated gastro-intestinal conditions. Int J Food Microbiol. 2010; 42:185–189. 10.1016/j.ijfoodmicro.2010.06.022 20659775

[32] And CI, Kailasapathy K. Effect of co-encapsulation of probiotics with prebiotics on increasing the viability of encapsulated bacteria under *in vitro* acidic and bile salt conditions and in yogurt. J Food Sci 2005; 70: M18–M23.

[33] Ramirez A, Saldanha PH. Micronucleus investigation of alcoholic patients with oral carcinomas. Genet Mol Res. 2002; 30: 246–260. 14963832

[34] Castelli E, Hrelia P, Maffei F, Fimognari C, Foschi FG, Caputo F et al Indicators of genetic damage in alcoholics: reversibility after alcohol abstinence. Hepatogastroenterol. 1999; 46: 1664–1668. 10430317

[35] Huttner E, Gotze A, Nikolova T. Chromosomal aberrations in humans as genetic endpoints to assess the impact of pollution. Mutat Res. 1999; 445: 251–257. 10575434 10.1016/s1383-5718(99)00130-8

[36] Lu YC, Yeh WC, Ohashi P. LPS/TLR4 signal transduction pathway. Cytokine. 2008; 42: 145–151. 10.1016/j.cyto.2008.01.006 18304834

[37] Wheeler MD. Endotoxin and Kupffer cell activation in alcoholic liver disease. Alcohol Res Health. 2003; 27: 300–306. 15540801 PMC6668869

[38] Schwabe RF, Seki E, Brenner DA. Toll-like receptor signaling in the liver. Gastroenterol. 2006; 130: 1886–1900. 10.1053/j.gastro.2006.01.038 16697751

[39] Hegazy SK, El-Bedewy M.M. Effect of probiotics on pro-inflammatory cytokines and NF-κB activation in ulcerative colitis. World J Gastroenterol. 2010; 16: 4145–4151. 10.3748/wjg.v16.i33.4145 20806430 PMC2932917

[40] Han X, Shen T, Lou H. Dietary polyphenols and their biological significance. Int J Mol Sci 2007; 8: 950–988.

[41] Barrie AM, Scott EP. The interleukin-12 family of cytokines: Therapeutic targets for inflammatory disease mediation. Clinical Appl Immunol Rev. 2005; 5: 225–240.

[42] Finamore A, Roselli M, Imbinto A, Seeboth J, Oswald IP, Mengheri E. *Lactobacillus amylovorus* inhibits the TLR4 inflammatory signaling triggered by enterotoxigenic *Escherichia coli* via modulation of the negative regulators and involvement of TLR2 in intestinal Caco-2 cells and pig explants. Plos One. 2014; 9: e94891 10.1371/journal.pone.0094891 24733511 PMC3986366

