# Supplementary material for: Better Management of Alcohol Liver Disease Using a ‘Microstructured Synbox’ System Comprising L. plantarum and EGCG
Source: PLoS One. 2017 Jan 6;12(1):e0168459. doi: 10.1371/journal.pone.0168459 (PMC5217831; doi:10.1371/journal.pone.0168459)
Supplement: S1 Table — (DOCX) [file pone.0168459.s001.docx]

| **Probiotic- EGCG Alginate beads** | | | | | | | | | | | |
| --- | --- | --- | --- | --- | --- | --- | --- | --- | --- | --- | --- |
| **Time** | **Simulated gastric fluid** | | | | | | **Simulated intestinal fluid** | | | | |
|  | 0 hour | 1 hour | | 2 hour | | 4 hour | 5 hour | | 6 hour | | % |
| **Log CFU**  ***L. plantarum*** | 8.85±0.30 | 8.34±0.13 | | 8.00±0.15 | | 7.58±0.18 | 6.96±0.14 | | 6.39±0.05 | | 72.22% |
| **Probiotic Alginate beads** | | | | | | | | | | | |
| **Time** | **Simulated gastric fluid** | | | | | | **Simulated intestinal fluid** | | | | |
|  | 0 hour | 1 hour | | 2 hour | | 4 hour | 5 hour | | 6 hour | | % |
| **Log CFU**  ***L. plantarum*** | 8.59±0.39 | 8.14±0.23 | | 7.5±0.43 | | 7.2±0.38 | 6.26±0.34 | | 5.97±0.09 | | 69.4% |
| **Probiotic- EGCG Alginate beads** | | | | | | | | | | | |
| **Time** | **0.3 % Bile Salts** | | | | | | | | | | |
|  | Initial Count | | 1 hour | | 2 hour | 3 hour | | 4 hour | | % | |
| **Log CFU**  ***L. plantarum*** | 8.95±0.29 | | 8.75±0.11 | | 8.35±0.27 | 8.00±0.20 | | 7.75±0.21 | | 86.59% | |
| **Probiotic Alginate beads** | | | | | | | | | | | |
| **Time** | **0.3 % Bile Salts** | | | | | | | | | | |
|  | Initial Count | | 1 hour | | 2 hour | 3 hour | | 4 hour | | % | |
| **Log CFU**  ***L. plantarum*** | 8.74±0.37 | | 8.25±0.18 | | 7.85±0.54 | 7.74±0.16 | | 7.55±0.67 | | 86.38 % | |

**S1 Table- Log_10_ CFU of *L. plantarum* entrapped in probiotic- EGCG beads in SGF, SIF and Bile salts.**
