# Supplementary material for: Better Management of Alcohol Liver Disease Using a ‘Microstructured Synbox’ System Comprising L. plantarum and EGCG
Source: PLoS One. 2017 Jan 6;12(1):e0168459. doi: 10.1371/journal.pone.0168459 (PMC5217831; doi:10.1371/journal.pone.0168459)
Supplement: S2 Table — (DOCX) [file pone.0168459.s002.docx]

|  | **Control** | **Alcohol group** | **Alcohol+ Free Probiotic-EGCG group** | **Alcohol+ Encapsulated Probiotic-EGCG group** | **Free Probiotic-EGCG group** | **Encapsulated Probiotic-EGCG group** |
| --- | --- | --- | --- | --- | --- | --- |
| **ALT (IU/L)** | 41.45± 15.45 | 104.3± 17.56* | 48.72± 18.46 | **43.34±16.44*^#^** | 62.67± 23.86 | 42.65± 17.33 |
| **AST (IU/L)** | 270.34 ± 5.6 | 281.45± 21.40* | 273.25± 29.38 | **269.65± 39.40*^#^** | 276.28± 43.75 | 275.72± 21.19 |
| **ALP (IU/L)** | 160.45± 23.4 | 259.4±1 16.4* | 156.33± 79.16 | **163.21± 11.23*^#^** | 191.18± 32.71 | 164.24± 28.15 |

**S2 Table- Effect of free probiotic and encapsulated probiotic-EGCG on hepatic markers in the serum of control and alcohol-administered rats.** Values are represented as mean ± S.D. of eight different observations. *p<0.05 vs control, free Pro-EGCG group, # p<0.05 vs alcohol group.
