# Supplementary material for: Better Management of Alcohol Liver Disease Using a ‘Microstructured Synbox’ System Comprising L. plantarum and EGCG
Source: PLoS One. 2017 Jan 6;12(1):e0168459. doi: 10.1371/journal.pone.0168459 (PMC5217831; doi:10.1371/journal.pone.0168459)
Supplement: S1 Text — (DOCX) [file pone.0168459.s003.docx]

Intestinal permeability in the rat was determined by the method of Noth et al. using two non-metabolized sugars, lactulose (Himedia, India) and mannitol (Himedia, India). 300mg of lactulose and 200 mg mannitol were dissolved in 20 ml distilled water. After a fasting period of 12 hours, control rats, alcoholic rats and treated animals received 1 ml of the lactulose/mannitol solution by orogastral tube. One hour after feeding, blood was taken from the retro orbital route. Serum sample was deproteinated and two micro litre of the prepared sample was spotted on the TLC plates using Camag Automatic TLC sampler 4. The solvent system used was ethyl acetate: glacial acetic acid: water (3:2:2) with the detection system 10% H_2_SO_4_ in ethanol with heating of plates at 110°C, for 5 minutes. The TLC plates were then scanned at 455nm (Camag TLC Scanner 3). WinCat software was used for analysis of spots. The concentration of lactulose and mannitol was measured by comparing with the standards of lactulose and mannitol run on separate HPTLC plates.

**S1 Text- Measurement of intestinal permeability**
