# Supplementary material for: Better Management of Alcohol Liver Disease Using a ‘Microstructured Synbox’ System Comprising L. plantarum and EGCG
Source: PLoS One. 2017 Jan 6;12(1):e0168459. doi: 10.1371/journal.pone.0168459 (PMC5217831; doi:10.1371/journal.pone.0168459)
Supplement: S2 Text — (DOCX) [file pone.0168459.s004.docx]

Micronuclei analysis was done by the method of Schmid [17]. For this assay, liver tissues were washed with chilled homogenizing buffer (24 mM Sodium-EDTA buffer pH 7.5, containing 75 mM of NaCl). 200 mg of tissue was homogenized in 5.0 ml of this buffer at 500 rpm for 30 seconds. The homogenates were then centrifuged, supernatant was removed and fresh homogenizing buffer (0.4 ml) was added to re-suspend the hepatocytes. Small drops of suspension prepared were put at one end of pre-cleaned, grease free microscopic slide and thin smooth film was prepared. The slides were air dried and then stained according to the following sequence: fixed in methanol for 10 min; stained in undiluted May-Grunwald solution for 90 seconds; washed with water; stained with Giemsa (1:10 diluted) for 15–20 min; rinsed twice in distilled water; blot dried with filter paper; back side of the slides cleaned with methanol; slides then cleared in xylene for 5 min, and finally mounted in DPX (Distyrene Plasticizer Xylene). Minimum of 100 cells were counted per sample for the presence of micronuclei using light microscope at 45x.

**S2 Text- Micronucleus Analysis method**
